# Effects of oxygen exposure on relative nucleic acid content and membrane integrity in the human gut microbiota

**DOI:** 10.7717/peerj.10602

**Published:** 2021-02-03

**Authors:** Mariia Taguer, Ophélie Quillier, Corinne F. Maurice

**Affiliations:** Department of Microbiology and Immunology, McGill University, Montreal, QC, Canada

**Keywords:** Gut microbiota, Flow cytometry, Bacterial physiology, Anaerobic, Bacterial activity, Bacterial diversity, Membrane integrity

## Abstract

While the diversity of the human gut microbiota is becoming increasingly well characterized, bacterial physiology is still a critical missing link in understanding how the gut microbiota may be implicated in disease. The current best practice for studying bacterial physiology involves the immediate storage of fecal samples in an anaerobic chamber. This reliance on immediate access to anaerobic chambers greatly limits the scope of sample populations that can be studied. Here, we assess the effects of short-term oxygen exposure on gut bacterial physiology and diversity. We use relative nucleic acid content and membrane integrity as markers of bacterial physiology, and 16S rRNA gene amplicon sequencing to measure bacterial diversity. Samples were stored for up to 6 h in either ambient conditions or in anoxic environments created with gas packs or in an anaerobic chamber. Our data indicate that AnaeroGen sachets preserve bacterial membrane integrity and nucleic acid content over the course of 6 h similar to storage in an anaerobic chamber. Short-term oxygen exposure increases bacterial membrane permeability, without exceeding inter-individual differences. As oxygen exposure remains an important experimental consideration for bacterial metabolism, our data suggest that AnaeroGen sachets are a valid alternative limiting loss of membrane integrity for short-term storage of samples from harder-to-access populations.

## Introduction

The human gut microbiota is increasingly studied due to its widespread implications in human health. This collection of microorganisms and their genes are involved in nutrient acquisition and digestion, pathogen exclusion, and development of the immune system (Gilbert et al., 2016). Disease-specific shifts in gut microbial communities have been correlated to many conditions from autism to *Clostridioides difficile* infection, with causation only shown in a subset of conditions (Gilbert et al., 2016). Extensive sequencing efforts have characterized gut bacterial diversity in a variety of human and non-human populations, describing how this bacterial diversity changes over time and in response to different perturbations such as dietary changes, antibiotics exposure, and disease (Gilbert et al., 2016; David et al., 2014; Dethlefsen et al., 2008). These studies typically highlight the high diversity of bacterial species or strains that stably colonize healthy individuals over long periods of time, with variations in relative abundances (Faith et al., 2013). At the functional level, this bacterial diversity provides high metabolic redundancy maintained across healthy individuals (Huttenhower et al., 2012). In disease, functional changes have been more consistently observed and predictive of the diseased state than bacterial diversity alone (The Integrative HMP (iHMP) Research Network Consortium, 2019). Thus, understanding how the gut microbiota is involved in disease requires a better understanding of how bacterial activity and metabolism change in addition to changes in bacterial diversity. Studies of bacterial diversity rely heavily on sequencing, providing a snapshot of the DNA present in the sample. As such, sequencing does not provide information on the state of the bacteria (Emerson et al., 2017). Determining which bacteria are alive, dead, or damaged remains elusive through sequencing techniques alone. To characterize different states of bacteria being sampled, we turn to single-cell techniques such as physiological dyes and flow cytometry.

Specifically, to identify bacteria with disrupted membranes, we use the membrane exclusion dye propidium iodide (PI) (Sträuber & Müller, 2010). Metabolic activity can be broadly measured with a nucleic acid dye such as SYBR Green I (Bouvier, Del Giorgio & Gasol, 2007). This dye is used to differentiate complex microbial communities into high and low nucleic acid content bacteria. Extensive data demonstrate that relative nucleic acid content and membrane integrity are relevant to understand specific bacterial contribution to community measurements of metabolism. Indeed, in some ecosystems, bacteria with higher nucleic acid content (HNA), determined by the combined use of nucleic acid-staining dyes and flow cytometry, are metabolically more active than their lower nucleic acid content counterparts (LNA) (Servais et al., 2003; Morán et al., 2007; Wang et al., 2009; Lebaron et al., 2001). Monitoring changes in the proportion of HNA bacteria in drinking water facilities allowed for the rapid identification of possible contamination in the system by identifying flares in bacterial activity rather than focusing on contaminant DNA (Prest et al., 2013). In mouse models of colitis, Zimmerman and colleagues showed a dynamic cytometric fingerprint of the gut microbiota during disease progression (Zimmermann et al., 2016; Günther et al., 2012). These changes in bacterial physiology were concordant with changes in taxonomic composition, thereby demonstrating that flow cytometry could be an alternate approach to rapidly monitor changes in bacterial community structure (Zimmermann et al., 2016). The HNA and LNA populations have been identified in the human gut microbiota as well, and have been shown to contain the same bacterial taxa at different relative abundances and different levels of gene transcription (Maurice, Haiser & Turnbaugh, 2013; Peris-Bondia et al., 2011). This suggests that relative nucleic acid content is not a marker of taxonomic composition in the human gut microbiota and could be linked to transcriptional activity. However, the complex links between nucleic acid content and metabolism remain to be further studied and validated, and are outside the scope of this study.

Functional studies of the gut microbiota require fresh samples, as sample freezing or aerobic processing both significantly increase the number of cells with impaired membranes compared to sample processing in an anaerobic chamber (Maurice & Turnbaugh, 2013). Numerous studies have tested storage and transportation conditions on fecal bacterial diversity when collecting samples from field sites (Fouhy et al., 2015; Choo, Leong & Rogers, 2015), yet there is no equivalent for bacterial physiology. On-site stool donations and immediate processing in anoxic conditions preclude the functional study of more diverse human populations, including clinical populations by sampling at home or in remote locations, and thus greatly limit the scope of current studies. Previous studies have shown that oxygen exposure affects bacterial activity and diversity, with loss of bacterial viability within 24 h for human samples (Browne et al., 2016), and strong changes in gut bacterial diversity after 12 h in bat guano (Fofanov et al., 2018). Such studies highlight the importance of assessing the effects of short-term oxygen exposure on gut bacterial communities, allowing for sample collection and delayed transportation to the laboratory.

Gas packs that rapidly create anoxic conditions are currently used for short-term storage and transportation of certain bacterial clinical isolates from stool, but have yet to be validated for the gut microbiota (Holý & Chmelař, 2012). Focusing on relative nucleic acid content and membrane integrity, we aimed to determine the appropriate short-term storage conditions to analyze these specific aspects of gut bacterial physiology. Using fecal samples from healthy unrelated volunteers, we tested three different storage conditions: anoxic environment created by an anaerobic gas pack (GP), anoxic environment of an anaerobic chamber (AN), and aerobic storage (AE), over 6 h. Our data show individualized responses of bacterial communities to the different storage methods, but indicate that short-term storage of samples with GP is an adequate alternative to immediate storage in an anaerobic chamber (AN). This alternative would allow for increased functional studies such as culture-based assays of gut microbial communities in a variety of systems.

## Methods

### Sample collection

Human studies were performed with approval of the McGill Ethics Research Board (REB #A04-M27-15B). Nineteen healthy, unrelated individuals who had not taken antibiotics in the past 6 months and had not been diagnosed with a gastro-intestinal condition provided fecal samples on site to establish inter-individual variation in the proportion of HNA and PI^+^ bacteria. Eleven donors provided samples for the anaerobic chamber, GP, and aerobic comparisons. Enrolled volunteers provided their written consent to participate in the study. Samples were immediately placed in the anaerobic chamber (Coy Laboratory Products, 5% H_2_, 20% CO_2_, 75% N_2_) unless otherwise noted. Sample preparation and staining were performed in the anaerobic chamber; flow cytometry acquisition was performed aerobically. With each donation metadata was collected including: age, weight, height, recent international travel, diet log for the past 48 h, typical dairy, caffeine, and soda consumptions, as well as last antibiotics used.

### Aerobic and anaerobic conditions

Three subsamples (1–4 g) of each fecal sample were taken and stored either in oxic conditions (AE), anoxically with the use of an AnaeroGen Gas pack (Oxoid AN0025A) (GP), or in an anaerobic chamber (Coy Anaerobic Systems, 5% H_2_, 20% CO_2_, 75% N_2_), (AN) for 6 h at room temperature. After 6 h, samples were processed and analyzed as. The gas pack setup is as follows: 16 h before sample arrival, one gas pack is placed inside a ziplock bag with an oxygen indicator dry strip (BD BBL) to ensure full oxygen removal as evidenced by a color change on the oxygen indicator. In addition to the gas pack, the ziplock bag also contained a 50 mL conical tube with a gas exchange lid (Bio-reaction tube, Ultident cat# 229475). Immediately after sample collection, a portion of the sample is placed inside the 50 mL tube, which is quickly placed inside the bag with a new gas pack and sealed (<1 min). The absence of oxygen was confirmed visually with the oxygen indicator strip. Lastly, part of the sample was immediately stored at −80 °C.

### Gut microbiota sample preparation

A portion (1–4 g) of the fresh fecal sample was diluted 1/10 w/v in pre-reduced PBS (rPBS) containing 1 ug.ml^−1^ resazurin sodium salt and 1 mg.ml^−1^ L-Cysteine (final concentrations). Samples were thoroughly vortexed to completely solubilize them, and were then centrifuged at 700×*g* for 1 min to pellet large organic debris. One milliliter of supernatant was washed 3 times with rPBS by centrifuging for 3 min at 6,000×*g*, removing supernatant, and resuspending with fresh rPBS. Each sample was then diluted 1/10,000 v/v in rPBS for staining and flow cytometry acquisition. Staining was performed in the dark with 1X SYBR Green I (Invitrogen, Carlsbad, CA, USA) for 15 min, or PI (Sigma-Aldrich, St. Louis, MO, USA) at 0.04 mg.ml^−1^ for 10 min with three technical replicates for each stain (Maurice & Turnbaugh, 2013).

### Flow cytometry acquisition

Samples were acquired on a BD FACSCalibur with 488 nm laser (15 mW) and FL1 and FL2 detection filters for SYBR Green I and PI capture, respectively, or on a BD FACSCanto II equipped with a 488 nm laser (20 mW) and 530/30 and 585/42 detection filters. Rainbow fluorescent particles of 3.0–3.4 μm (BD Biosciences, San Jose, CA, USA) were added to each sample before acquisition in sufficient volume (10–30 μL) to acquire bead events equivalent to ~1% of total events. Rainbow fluorescent particle concentration and total counts were determined after acquisition with CountBright Absolute Counting Beads, 7 μm (Life Technologies, Carlsbad, CA, USA) as per previous studies (Maurice & Turnbaugh, 2013). Flow cytometers are calibrated daily, and the use of Rainbow beads (BD Biosciences, San Jose, CA, USA) allows for internal calibration across days. Flow cytometry analysis was performed with FlowJo 10.2.

### Statistical analysis

Statistical analysis was performed with GraphPad Prism v7.0 for MacOS X. Friedman non-parametric tests correcting for multiple comparisons were used to analyze differences in active cells and cells with loss of membrane integrity in the GP or AN conditions for the 6-h time course. One-way ANOVA correcting for multiple comparisons with post-hoc Tukey’s test was used to analyze changes in the proportion of active cells and cells with loss of membrane integrity in the studies comparing GP and AN conditions to AE.

### DNA extraction and 16S gene amplicon bioinformatics analysis

Samples were stored at −80 °C until DNA extraction. Samples were extracted with the Qiagen Powersoil kit as per the manufacturer’s instructions. The V4 hypervariable region was amplified with the 515F/806R primers (Caporaso et al., 2012). Bioinformatics was performed with the quantitative insights into microbial ecology (QIIME2) platform (Bolyen et al., 2018). Trimming, alignment of paired end reads, and quality filtering was performed with DADA2 (Callahan et al., 2016). Taxonomic alignment was performed with a pre-trained Naives Bayes classifier using SILVA 132 database on 99% OTUs. Alpha diversity was assessed by Faith’s phylogenetic diversity using a paired one-way ANOVA with Dunnett’s multiple comparisons test, after normalizing to a subsampling depth of 58,733 reads per sample. Alpha rarefaction curves were generated to confirm this subsampling depth was adequate in retrieving sequence diversity. Beta diversity was assessed by weighted UniFrac distances using pairwise PERMANOVA with 999 permutations to test for significance. The Mantel test, using Spearman’s rank correlation was performed on the weighted UniFrac matrix on the initial samples, compared to the proportion of HNA and PI^+^ bacteria after oxygen exposure with 999 permutations to test for significance. Alpha diversity correlation to the proportion of HNA and PI^+^ bacteria after oxygen exposure was tested on the Shannon’s diversity index using Spearman’s rank correlation.

### Data availability

Bacterial 16S rRNA gene sequencing data can be accessed on the SRA database, accession number PRJNA656580. Flow cytometry files can be accessed on the Flow Repository, under ID FR-FCM-Z3YJ and FR-FCM-Z323.

## Results

### Bacterial physiology varies across individuals but is stable over time within individuals

**Figure 1:**
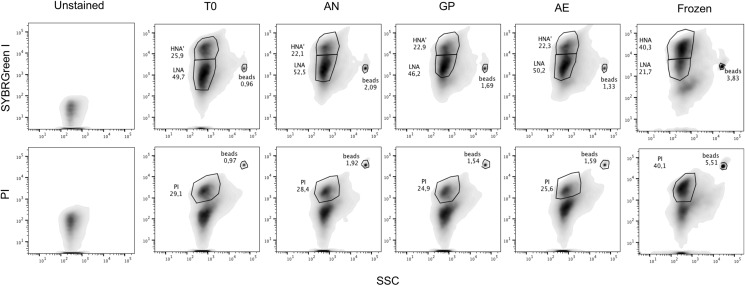
Representative flow cytometry plots after SYBR Green I and Propidium Iodide staining. One representative flow plot from each storage condition tested with each stain from one individual is shown, as well as a frozen sample. Gating is determined based off the unstained control. Top panels are SYBR Green I staining, with the HNA and LNA gates, bottom panels are with PI staining, with the PI^+^ cells gated and with unstained cells and debris below the gate. From left to right: Unstained control, initial timepoint (T0), anaerobic chamber (AN), gas pack (GP), aerobic storage (AE), and frozen sample. Fluorescent rainbow beads were added to each tube as an internal calibration and are annotated. Percentages of HNA, LNA, and PI^+^ cells, identified by FlowJo, are provided.

**Figure 2:**
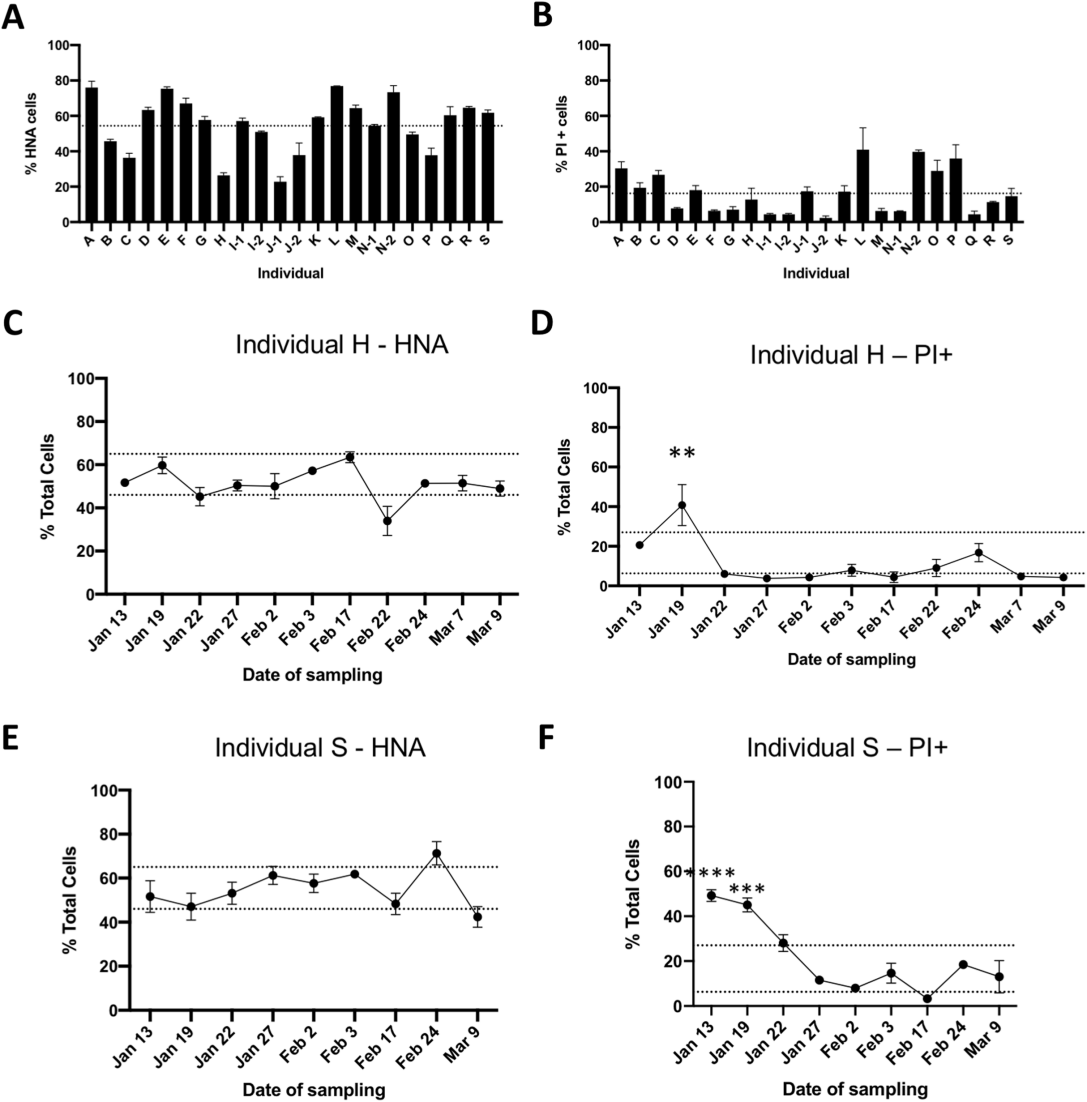
Flow cytometric analysis of gut bacterial physiology in healthy individuals. Fresh fecal samples were obtained from 19 healthy, unrelated individuals and stained with SYBR Green I to determine the proportions of HNA bacteria (A), or stained with PI to determine the proportions of bacteria with loss of membrane integrity (B). Dotted line represents the mean from all individuals, error bars represent standard deviation (SD) of three technical replicates. Individuals I, J, and N were sampled on two different days. To further determine temporal changes in the proportions of HNA and PI^+^ bacteria, fresh fecal samples were obtained from two individuals over the course of 8 weeks. Panels (C) and (E) follow HNA over time, and panels (D) and (F) follow PI^+^ over time in individuals H and S, respectively. Dotted lines represent the inter-quartile range based off all 19 individuals. Statistical analysis compared individual days to the average from all 19 individuals by two-way ANOVA, with Sidak’s test for multiple comparisons. *p < 0.05, **p < 0.01, and ***p < 0.001.

We determined the proportions of HNA and LNA bacteria through SYBR Green I staining, and the proportions of cells with impaired membranes through PI staining from fecal samples of 19 healthy unrelated individuals. Flow cytometry plots with representative gating based on unstained samples are depicted in Fig. 1. Male and female volunteers were aged between 19–67 years old, had a BMI between 17.9 and 30.4, and were all from the Montreal area (Table 1). The proportion of HNA cells averaged 56% ± 16% ranging from 23% to 77%, with significant differences between individuals (*p* < 0.0001, one-way ANOVA, Fig. 2A). The proportion of bacteria with loss of membrane integrity, as assessed by PI staining, ranged from 4.3% to 41%, averaging 17.18 ± 11.4% and was also significantly different across individuals (*p* < 0.0001, one-way ANOVA, Fig. 2B. Statistical analysis was performed on a per flow cytometer basis). As nucleic acid content has previously been associated to the size of the cells, we compared median SSC values between HNA and LNA and found that HNA had significantly higher SSC values than LNA cells (*p* < 0.0001, paired *t*-test, Fig. S1).

**Table 1:** Volunteer metadata. Metadata of all volunteers enrolled in the study.

Volunteer	Age	Sex	BMI
A	40	M	19.3
B	19	F	23.4
C	38	F	20.7
D	36	M	22.5
E	22	F	17.9
F	22	M	22.5
G	21	M	24.7
H	30	M	26.9
I	36	M	29.6
J	26	F	20.1
K	26	M	22.7
L	21	M	25.4
M	19	F	18.7
N	30	F	27.1
O	33	M	21.8
P	21	M	22.0
Q	19	M	19.7
R	67	M	30.4
S	25	M	23.5
Average ± SD	28.6 ± 11.5	32% (6) F 68% (13) M	23.1 ± 3.5
Range	19–67		17.9–30.4

To explore the variability within individuals, two volunteers provided weekly fecal samples for several weeks (*n* = 9 for volunteer S and *n* = 11 for volunteer H). The proportion of HNA bacteria in individual H (Fig. 2C) over time averaged 51.2 ± 7.8%, with only one timepoint outside the interquartile range based off the proportion of HNA of all individuals. Similarly for individual S, the proportion of HNA bacteria averaged 54.9 ± 8.9% with two timepoints outside the interquartile range (Fig. 2E). However, no timepoint was significantly different than the average proportion of HNA calculated across all 19 individuals. The proportion of PI^+^ bacteria were also stable over time, with an average of 11.16 ± 11.3% for individual H and 21.25 ± 16.2% for individual S, with one and two timepoints outside of the interquartile range, respectively, and were significantly different than the average proportion of PI^+^ bacteria across all 19 individuals (Two-way ANOVA, with Sidak’s test for multiple comparisons, Figs. 2D and 2F). Changes in physiological proportions could not be linked to volunteer metadata collected (see “Methods” for metadata acquired).

### Changes in bacterial physiology in response to oxygen exposure are individual-dependent

**Figure 3:**
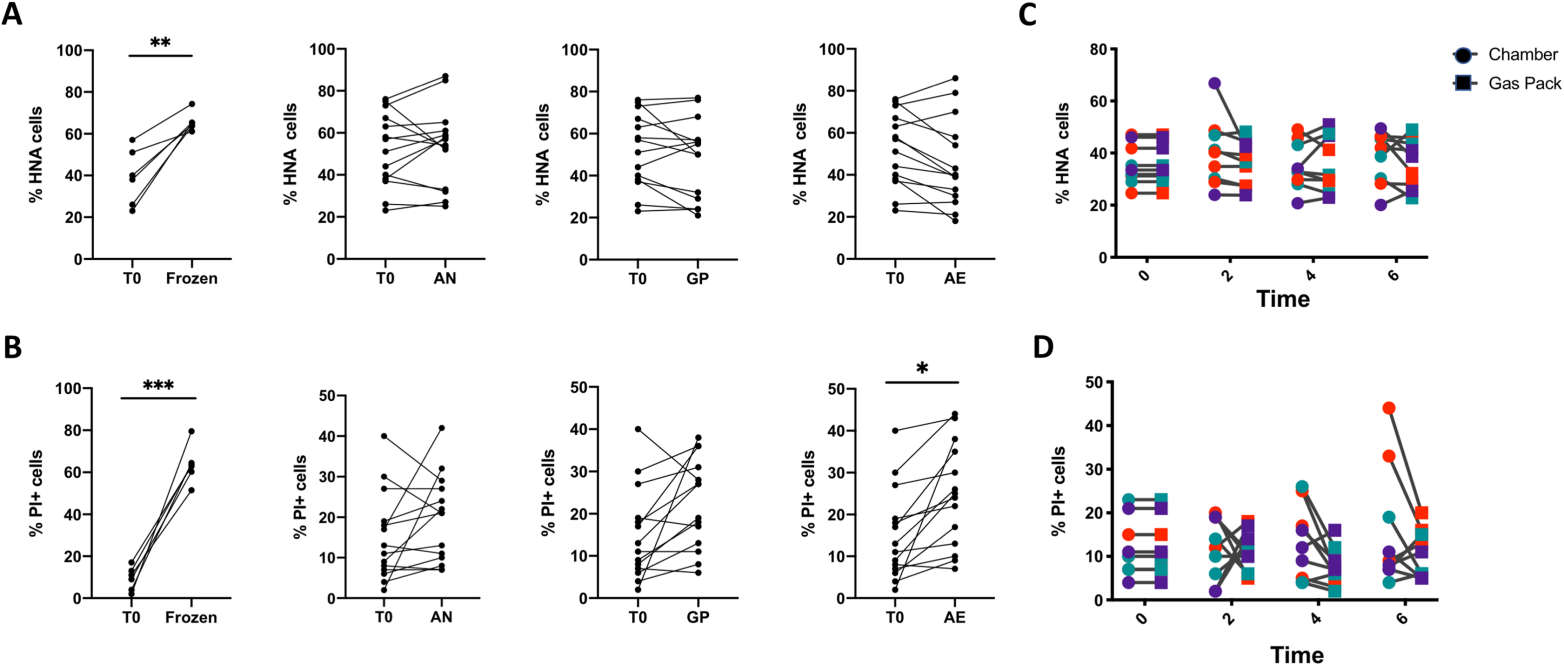
Analysis of the proportions of HNA and PI^+^ bacteria after 6 h of storage compared to the initial timepoint. (A) The proportion of HNA and (B) PI^+^ bacteria after various storage conditions compared to the initial fresh sample. From left to right: Fecal samples were stored at −80 °C (Frozen), in an anaerobic chamber (AN), with a gas pack to maintain anoxic conditions (GP), or left in oxic conditions (AE) for 6 h. Paired *t*-test with Holm-Sidak test for multiple comparisons (*N* = 11). (C) A time course was performed comparing AN to GP conditions in three individuals on three different days. Differences in the proportion of (C) HNA bacteria and (D) PI^+^ bacteria were assessed with repeated measures two-way ANOVA with Sidak’s correction for multiple comparisons (*N* = 9).

As shown previously, freezing samples significantly increased the proportion of HNA and PI^+^ bacteria (Figs. 3A and 3B), highlighting the importance of sample storage considerations when studying bacterial physiology. We then tested the effects of three 6-h storage conditions with increasing levels of oxygen exposure on bacterial physiology on all samples: aerobic conditions (AE), anoxic conditions created by GP, and anoxic conditions created by an anaerobic chamber (AN). Oxygen levels were qualitatively assessed with an oxygen indicator strip. Relative to the initial timepoint, the proportions of HNA and LNA bacteria were not significantly altered in any of the storage conditions (Fig. 3A), but bacterial membrane permeability significantly increased from 17.18% to 24.27% under aerobic conditions (*p* < 0.05, repeated measures one-way ANOVA, with Tukey’s test for multiple comparisons, Fig. 3B). There was a significant decrease in absolute cell abundances after 6 h, irrespective of oxygen exposure (Fig. S2). However, when considering only the 6-h timepoint, we report no significant difference in the proportions of HNA or PI^+^ bacteria, as well as total counts between samples stored in anoxic conditions in the chamber or under ambient aerobic conditions.

A two-way ANOVA analysis indicates that the source of variation in bacterial physiology over time and in response to oxygen exposure is largely driven by the individual. Indeed, changes in bacterial physiology in response to distinct levels of oxygen are smaller than those observed between individuals (Table 2). There is a statistically significant interaction between the effect of the individual and the oxygen exposure conditions on the proportions of HNA and PI^+^ bacteria, where the interaction explains 7.45% of the total variation for HNA, and 13.6% for PI^+^ (*p* < 0.0001 for both, ordinary two-way ANOVA). The variance between individuals accounts for 90% of the variation in the proportion of HNA bacteria, whereas the level of oxygen exposure explains only 0.99% of the variation in the proportion of HNA bacteria (*p* < 0.0001 for both, ordinary two-way ANOVA). Similarly for the proportion of PI^+^ bacteria, the differences between individuals explained 68.69% of the variation, while the level of oxygen exposure explained 5.04% of the variation (*p* < 0.0001 for both, ordinary two-way ANOVA).

**Table 2:** Effects of oxygen exposure on the proportions of HNA and PI^+^ bacteria. Results of 2-way ANOVA comparing the effects of oxygen exposure conditions across individuals on the proportions of HNA and PI^+^ bacteria.

	Individual		Condition		Interaction	
	% of total variation	*p*-value	% of total variation	*p*-value	% of total variation	*p*-value
**HNA**	90.03	<0.0001	0.9922	<0.0001	7.454	<0.0001
**PI**^**+**^	68.69	<0.0001	5.044	<0.0001	13.60	<0.0001

### Bacterial physiology does not change over the course of 6 h in anoxic conditions

As the GP and AN storage conditions led to similar bacterial physiology after 6 h, we further proceeded with a time course of bacterial physiology every 2 h for 6 h to explore more subtle dynamics between these two storage conditions (Figs. 3C and 3D). There were no significant differences in the proportions of HNA or PI^+^ cells between GP and AN storage conditions, whether considering all samples together or within each individual. Indeed, over the 6-h time course, the proportion of active HNA cells averaged 37.4% ± 10.0% of the whole community under AN conditions, and 36.0% ± 8.7% of the whole community under GP conditions. Similarly, the proportions of permeable PI^+^ bacteria averaged 13.1% ± 9.2% of the whole community under AN conditions, compared to 10.4% ± 5.3% of the whole community under GP conditions over the time course. This suggests that anoxic storage conditions, whether through an anaerobic chamber or the use of gas packs, maintain bacterial physiology over the course of 6 h.

### Short-term oxygen exposure does not alter bacterial diversity

**Figure 4:**
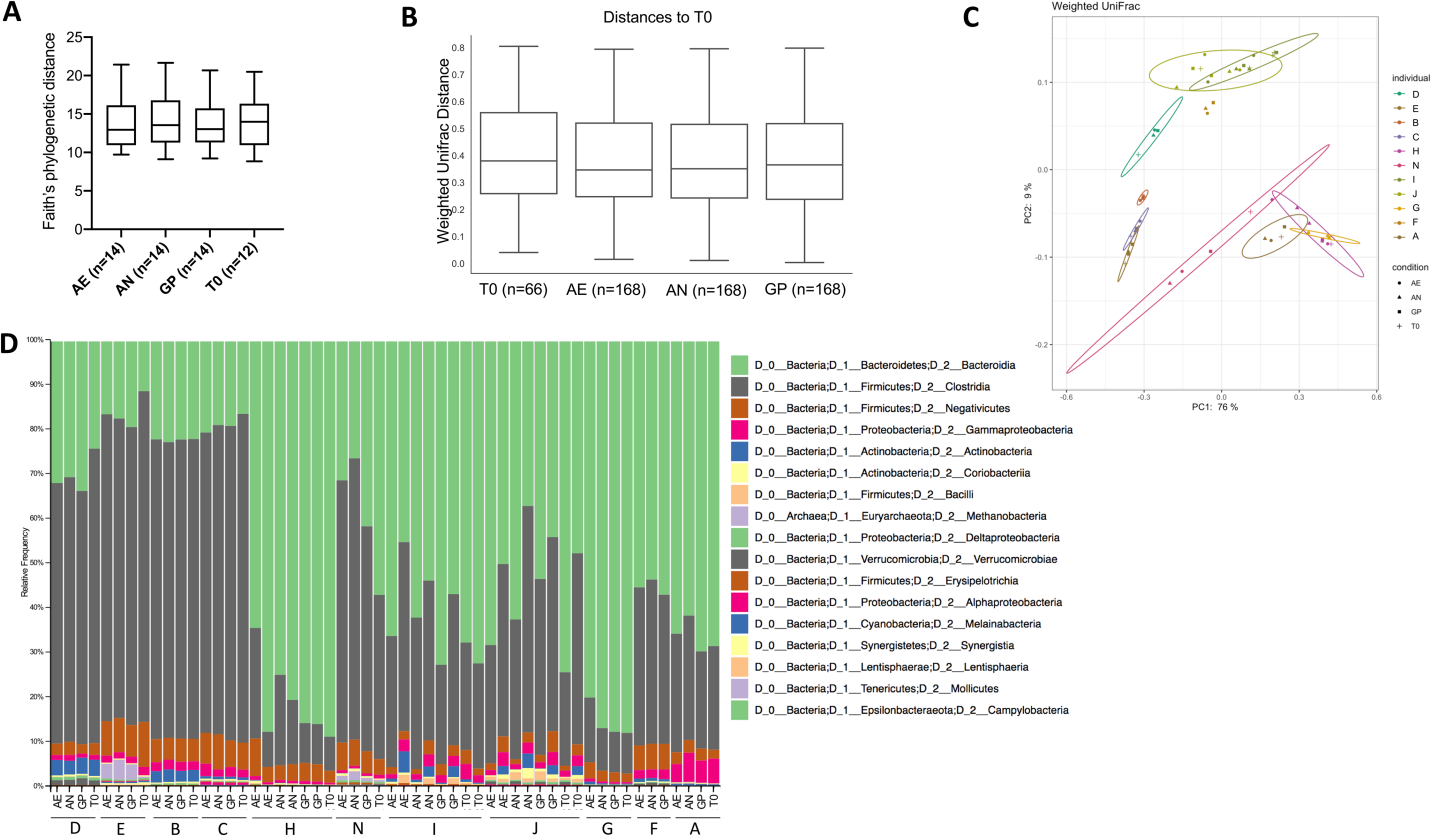
Bacterial diversity according to oxygen exposure conditions. (A) Alpha diversity calculated from Faith’s phylogenetic distances, with no significant differences between oxygen exposure conditions (paired one-way ANOVA with Dunnett’s multiple comparisons test). (B) Beta diversity between oxygen exposure conditions are not significantly different when comparing weighted UniFrac distances (PERMANOVA with 999 permutations, test statistic = 0.110997, *p*-value = 0.999). (C) Principal coordinate analysis of weighted UniFrac distances with the first two principal coordinates plotted on the *x*- and *y*-axis, respectively. Individuals are color coded, and oxygen exposure conditions are indicated by shape. (D) Relative abundance at the class level for each individual and storage method (T0, Baseline, AN, Anaerobic Chamber, GP, Anaerobic Gas Pack, AE, Aerobic). One to two samples were used per individual with *n* = 11 per oxygen exposure condition to determine bacterial diversity.

Community composition analysis was performed through 16S rRNA gene amplicon sequencing of the V4 region and analysis performed with quantitative insights into microbial ecology (QIIME2) version 2019.10 (Bolyen et al., 2019). A total of 6,924,735 demultiplexed sequences were obtained, with samples containing a range from 58,761 to 266,949 sequences. After quality filtering, there were a total of 6,654,667 sequences, ranging from 58,733 to 266,827 sequences per sample. Alpha diversity within each sample, as assessed by Faith’s phylogenetic diversity and a paired one-way ANOVA with Dunnett’s test for multiple comparisons, was not significantly different according to oxygen exposure conditions (Fig. 4A), but was significantly different according to the individual (*p* < 0.0001). Similarly, the beta diversity was significantly different between individuals (weighted UniFrac, *p* = 0.001, PERMANOVA, 999 permutations), but not according to the oxygen exposure conditions (Figs. 4B, 4C and 4D). Principle coordinate analysis (PCoA) plots of weighted UniFrac distances show clustering of individuals and not based on storage conditions (Fig. 4C), with 85% of the total variation explained in the first two axes.

Changes in gut bacterial physiology in response to oxygen exposure are not significantly correlated to the initial microbial community when comparing the diversity of the initial community to the proportion of HNA and PI^+^ bacteria (Fig. S3).

## Discussion

While the effects of oxygen exposure on gut bacterial diversity have been evaluated elsewhere (Fouhy et al., 2015; Choo, Leong & Rogers, 2015), its effects on bacterial physiology remain to be determined. Here, we explored the effects of short-term oxygen exposure that can result from sample transport to the laboratory, prior to the handling of samples in an anaerobic chamber. We monitored relative nucleic acid content and membrane integrity from fresh fecal samples immediately stored under anoxic conditions either in an anaerobic chamber (AN) or using GP, or stored aerobically (AE) for 6 h.

Overall, short-term oxygen exposure does not significantly alter the proportion of HNA bacteria, a possible proxy for metabolically active cells, yet causes a significant increase in the proportion of bacteria with an impaired membrane (PI^+^). Samples kept inside the anaerobic chamber or with an AeroGen gas pack for 6 h had similar levels of bacteria with loss of membrane integrity relative to the original fecal sample, and these levels remained comparable between these two storage conditions throughout the oxygen exposure time course. Both comparisons suggest that oxygen exposure is causing the increase in bacterial membrane permeability, and not the duration of storage. In addition, this validates the use of AnaeroGen gas packs to maintain sufficiently low oxygen levels such that bacterial physiology, specifically nucleic acid content and membrane integrity, of the fecal microbial community is not significantly altered. The significant interaction between the individual and oxygen levels on bacterial physiology suggests that the bacterial physiological response to oxygen exposure is individual-dependent. We saw no significant correlation between the initial taxonomic profile and the gut bacterial physiological response to oxygen exposure. As previous work has shown that the same bacterial taxa can be found in different physiological categories (Maurice, Haiser & Turnbaugh, 2013), the relationship between taxonomic composition and physiological changes in response to oxygen is not clear.

There were no significant changes in bacterial diversity as assessed through 16S rRNA gene amplicon sequencing, regardless of the length of exposure or storage condition. Other studies have reported an effect of oxygen on gut bacterial community composition, but after oxygen exposure times of up to 24 or 48 h in fecal samples (Choo, Leong & Rogers, 2015), or 4 days for mice exposed to hyperbaric conditions (Albenberg et al., 2014). In a study similar to ours, Chu et al. (2017) assessed the effects of oxygen exposure for 7 h, and compared processing samples in either oxic or anoxic conditions for fecal microbiota transplants. In this case, the authors saw decreases in bacterial abundance with increasing oxygen exposure, which we also observed, despite no link with the storage conditions and resulting oxygen levels in our case. The authors also reported minimal changes in bacterial composition through 16S rRNA gene analysis of the whole community. However, when they proceeded with the removal of dead bacteria prior to sample sequencing, they found differences in bacterial diversity due to oxygen exposure during sample processing (Chu et al., 2017). Thus, our sequencing data are in agreement with the limited differences seen from anoxic processing without prior removal of dead bacteria. Yet, as we report an increase in the proportion of bacteria with loss of membrane integrity upon oxic storage conditions, it is likely that we would also observe an altered community if we were to remove this significant proportion of oxygen-damaged bacteria from our samples.

While we saw no differences in bacterial physiology or diversity with lower levels of oxygen from the use of the anaerobic gas pack, it is well known that different bacteria in the gut have varying degrees of oxygen sensitivity. For example, cultures of *Eubacterium*, *Coprococcus*, and *Peptostreptococcus* lost 50% cell viability after just 5 min of exposure to ambient oxygen (Brusa et al., 1989). As such, studies focusing on specific bacterial members of the community should validate the gas pack for their specific species of interest.

Physiological staining to monitor unique physiological clades of bacteria has been used in a variety of systems, from monitoring drinking water, the progression of colitis, to the effectiveness of wastewater treatment (Prest et al., 2013; Zimmermann et al., 2016; Günther et al., 2012). While the proportions of HNA and LNA to study relative bacterial metabolic activity have been widely used (Peris-Bondia et al., 2011; Maurice & Turnbaugh, 2013; Jellett et al., 1996), the mechanisms defining this distribution in the gut are still largely unknown. Therefore, the specific changes that occur when HNA and LNA populations change, while suggestive of overall community metabolism and physiology, remain elusive. Nucleic acid content has been linked with contrasting results to bacterial metabolism (Longnecker, Sherr & Sherr, 2005; Zubkov et al., 2001), genome and bacterial size, and to taxonomy (Bouvier, Del Giorgio & Gasol, 2007; Wang et al., 2009; Zimmermann et al., 2016; Proctor et al., 2018) according to the bacteria and ecosystem studied. LNA bacteria isolated from an aquatic system remained LNA in enrichment cultures, suggesting that LNA could be a taxonomic characteristic rather than a metabolic phenotype (Wang et al., 2009). In other studies, when the taxonomic distribution of HNA and LNA were similar, their cell-specific leucine incorporation rates were also similar, suggesting that differences in nucleic acid content were not due to metabolic activity as defined by protein production (Longnecker, Sherr & Sherr, 2005). In the human gut, the HNA and LNA fractions contain the same bacteria present at different relative abundances, suggesting here that nucleic acid content is not a taxonomic characteristic (Maurice, Haiser & Turnbaugh, 2013; Peris-Bondia et al., 2011). Using an independent metatranscriptomics approach, this study also identified the most abundant HNA bacteria as having higher levels of transcriptional activity (Maurice, Haiser & Turnbaugh, 2013). Finally, nucleic acid content could not be correlated to genome size, as suggested in other systems (Proctor et al., 2018), as Clostridiales tend to have smaller genomes than Bacteroidales. Collectively, these data support a link between nucleic acid content and metabolism in the human gut that remains to be further defined. While we have previously shown no difference in cell size between HNA and LNA bacteria in the human gut (Maurice, Haiser & Turnbaugh, 2013), here we found significant differences in side scatter measurements, a marker of cell size, between HNA and LNA fractions. This suggests a correlation between cell size and nucleic acid content that is worth further investigation (Fig. S1).

The use of PI to monitor cells with loss of membrane integrity has also been optimized for whole communities and bacterial isolates, including representatives of the five major phyla found in the human gut, as well as the mosquito midgut microbiota (Maurice & Turnbaugh, 2013; Habtewold, Duchateau & Christophides, 2016). Considering the known sensitivity of obligate anaerobes to oxygen (Patel, Roth & Agnew, 2010), the relatively rapid, but mild, increase in bacteria with loss of membrane integrity upon oxygen exposure seen in this study would be indicative of cell damage. As we did not separate the bacterial cells from the fecal matrix when testing these storage conditions, it is possible that the fecal matrix could protect the bacteria from more severe oxygen damage, as shown elsewhere (Oliver et al., 2006). Gut luminal contents have been shown to consume oxygen through both a microbiota dependent and independent manner (Friedman et al., 2018), possibly through the oxidation of phospholipids which was shown to reduce oxygen levels from 40 mmHg to near 0 within 6 h.

The sample storage, preparation, and flow cytometry setup performed in this study is amenable to other physiological dyes and other single-cell methods. Many fluorescent dyes indicative of cell physiology have been validated with isolates and complex communities, allowing researchers to determine cell size, shape, intracellular density, membrane polarity and damage, nucleic acid content, as well as various enzymatic functionalities practically instantly (Maurice & Turnbaugh, 2013; Wang et al., 2010; Koch et al., 2013). For example, performing fluorescently activated cell sorting and subsequent 16S rRNA amplicon sequencing (FACSeq) on these physiologically distinct fractions would provide us with more detailed information as to whether bacteria are changing their metabolism in response to oxygen exposure; help detail specifically which bacteria are being damaged by oxygen; and if their removal would alter the diversity of the overall community (Maurice, Haiser & Turnbaugh, 2013; Maurice & Turnbaugh, 2013).

Overall, our data indicate that changes in relative nucleic acid content and membrane integrity to oxygen exposure are individual-dependent. While short-term oxygen exposure causes a minor increase in bacterial membrane permeability, there are no significant changes in nucleic acid content or bacterial community diversity. Altogether, the effects of short-term storage under various levels of oxygen within individuals leads to minor variation relative to inter-individual differences in these two parameters of bacterial physiology. Despite our low sample size, the small but significant increase in bacterial permeability we report supports the notion that short-term oxygen exposure should be limited, and that gas packs creating anoxic conditions provide an adequate alternative to maintain at least certain aspects of bacterial physiology for up to 6 h. Going forward, the storage conditions such as oxygen exposure are important considerations when studying bacterial physiology and metabolism, and should not hinder the study of more diversified populations.

## Conclusions

In this study, we set out to explore adequate alternatives to short-term storage of fecal samples to study relative nucleic acid content and membrane integrity, two markers of bacterial physiology. Relative nucleic acid content and membrane integrity high levels of inter-individual variation, but are stable over time within individuals. Fecal samples exposed to ambient levels of oxygen had increased levels of bacteria with impaired membranes, with no changes in relative nucleic acid content. AnaeroGen gas packs provided sufficient oxygen removal as the proportions of bacteria with loss of membrane integrity or active bacteria, as determined by relative nucleic acid content, did not change.

## Supplemental Information

10.7717/peerj.10602/supp-1 Supplemental Information 1 Raw data for Figure 2, panels A and B, acquired on the Canto flow cytometer. Raw data for Flow cytometry cell counts for individuals A-G (Figs. 2A and 2B) obtained after acquisition on the Canto flow cytometer. Sample label, mean fluorescence and scatter, gated counts for cells and reference beads, volumes of beads and sample are all provided. Cell abundances are obtained using the gated counts on FlowJo, reference beads concentration, and sample volume. Proportions of HNA, LNA, and Pi cells are calculated relative to the total cell counts obtained by SybrGreen staining. Concentration of reference beads for each acquisition date are found in rows 199-203. Data used in Figures are in columns X (%HNA) and P (Pi+).

10.7717/peerj.10602/supp-2 Supplemental Information 2 Raw data for Figure 2, panels A-B, acquired on the Calibur flow cytometer. Raw data obtained after acquisition on the Calibur flow cytometer for 9 of the 19 individuals in Figure 2 A and B. Fresh fecal samples were immediately placed in the anaerobic chamber, prepared as discussed in the methods, and stained with either Sybr Green I or with PI. Each sample was stained and acquired in triplicate, with values averaged. Data presented in the manuscript correspond to the averages. The letter codes for each individual is the same as in the Figure 1. Sample label and gated counts for cells and reference beads are provided. Cell abundances are obtained using the gated counts on FlowJo, reference beads concentration, and sample volume. The columns contain the counts for each gate (beads, HNA, LNA, PI), and the rows represent the different samples with the first letter representing the individual, followed by the stain, and by the staining techical replicate.Proportions of HNA, LNA, and Pi cells are calculated relative to the total cell counts obtained by SybrGreen staining.

10.7717/peerj.10602/supp-3 Supplemental Information 3 Raw data for Figure 2, panels C to F. Raw data of longitudinal study of individual H. Fresh fecal samples were immediately placed in an anaerobic chamber twice a week for 2 months, to assess the intra-individual variation over time in the proportions of HNA and PI+ cells in the human gut microbiota. Raw data from the gates imported from FlowJo are noted, each column is a unique gate, and rows are samples over time.

10.7717/peerj.10602/supp-4 Supplemental Information 4 Raw data for Figure 3, panels A-B. Cell counts, including calculations based off bead concentrations (obtained in Sheet "Beads") obtained by flow cytometry. The T0 values for individuals H, I, and J are also incorporated in Figure 2A, B. Data used in figures is found in rows 17 to 91 in columns W (%HNA), Z (%LNA) and rows 95-169 in column I (PI+); we used the average values obtained from the technical replicates. A is for Aerobic storage; CH is Chamber, and GP is Gaspack. T6 is the 6 h timepoint.

10.7717/peerj.10602/supp-5 Supplemental Information 5 Raw data for Figure 3, panels A-B. Raw data imported from FlowJo for each gate on each sample. Each gate is noted in columns C-F, followed by dilution factors and volumes required to determine cell abundances.

10.7717/peerj.10602/supp-6 Supplemental Information 6 Raw data and Flow cytometry cell counts for Figure 3 C, D. Sample codes in column A are labeled as follows: timepoint - one letter to identify the individual - storage condition (C for Chamber, A or G for anaerobic gas pack) - the stain - the technical replicate - the date for individuals A-G (Fig 2A, B) obtained after acquisition on the Canto flow cytometer. Sample label, mean fluorescence and scatter, gated counts for cells and reference beads, volumes of beads and sample are all provided. Cell abundances are obtained using the gated counts on FlowJo, reference beads concentration (noted on sheet "Beads concentration"), and sample volume. Proportions of HNA, LNA, and Pi cells are calculated relative to the total cell counts obtained by SybrGreen staining. Rows highlighted in yellow correspond to samples where we averaged the bead counts from the other two technical replicates due to a technical issue during acquisition (clog, bubble, or beads that were too concentrated). Values in column V and AG were used as data points in the figures.

10.7717/peerj.10602/supp-7 Supplemental Information 7 Median SSC values between HNA and LNA cells. Left: Acquired on the Calibur (N=28); Right: Acquired on the Canto (N= 33). Statistical significance was assessed as a paired t-test, error bars represent SD. *** P<0.0001.

10.7717/peerj.10602/supp-8 Supplemental Information 8 Absolute cell abundances in each oxygen exposure condition. Statistical significance was assessed with repeated measures one way ANOVA with Dunnet’s multiple comparisons test. Paired samples are connected by a line between oxygen exposure conditions (n=8). * P<0.05.

10.7717/peerj.10602/supp-9 Supplemental Information 9 The diversity of the initial community does not reflect the proportion of HNA or PI^+^ bacteria after oxygen exposure for 6 hours. Spearman correlation between the weighted UniFrac distances of the initial population to the proportion of HNA (left) or PI^+^ (right) bacteria after oxygen exposure (N = 10 individuals, 3 oxygen exposure conditions each, pairwise comparisons).

## Additional Information and Declarations

### DNA Deposition

The following information was supplied regarding the deposition of DNA sequences: Bacterial 16S rRNA gene sequencing data is available at the SRA database: PRJNA656580.

### Data Availability

The following information was supplied regarding data availability: Raw numeric data for flow cytometry (Figs. 1–3) are available in the Supplemental Files. Flow cytometry files can be accessed on the Flow Repository: FR-FCM-Z3YJ and FR-FCM-Z323.

## References

[ref-1] Albenberg L, Esipova TV, Judge CP, Bittinger K, Chen J, Laughlin A, Grunberg S, Baldassano RN, Lewis JD, Li H, Thom SR, Bushman FD, Vinogradov SA, Wu GD (2014). Correlation between intraluminal oxygen gradient and radial partitioning of intestinal microbiota. Gastroenterology.

[ref-2] Bolyen E, Dillon M, Bokulich N, Abnet C, Al-Ghalith G, Alexander H, Alm E, Arumugam M, Asnicar F, Bai Y, Bisanz J, Bittinger K, Brejnrod A, Brislawn C, Brown T, Callahan B, Chase J, Cope E, Dorrestein P, Douglas G, Durall D, Duvallet C, Edwardson C, Ernst M, Estaki M, Fouquier J, Gauglitz J, Gibson D, Gonzalez A, Gorlick K, Guo J, Hillmann B, Holmes S, Holste H, Huttenhower C, Huttley G, Janssen S, Jarmusch A, Jiang L, Kaehler B, Keefe C, Keim P, Kelley S, Knights D, Koester I, Kosciolek T, Kreps J, Lee J, Ley R, Liu Y-X, Loftfield E, Lozupone C, Maher M, Marotz C, Martin B, McDonald D, McIver L, Melnik A, Metcalf J, Morgan S, Morton J, Navas-Molina J, Orchanian S, Pearson T, Peoples S, Petras D, Pruesse E, Rivers A, Robeson M, Rosenthal P, Segata N, Shaffer M, Shiffer A, Sinha R, Spear J, Swafford A, Thompson L, Torres P, Trinh P, Tripathi A, Turnbaugh P, Ul-Hasan S, Vargas F, Vogtmann E, Walters W, Wan Y, Wang M, Warren J, Weber K, Willis A, Zaneveld J, Zhang Y, Zhu Q, Knight R, Caporaso G (2018). QIIME 2: reproducible, interactive, scalable, and extensible microbiome data science. PeerJ Preprints.

[ref-3] Bolyen E, Dillon M, Bokulich N, Abnet C, Al-Ghalith G, Alexander H, Alm E, Arumugam M, Asnicar F, Bai Y, Bisanz J, Bittinger K, Brejnrod A, Brislawn C, Brown T, Callahan B, Chase J, Cope E, Dorrestein P, Douglas G, Durall D, Duvallet C, Edwardson C, Ernst M, Estaki M, Fouquier J, Gauglitz J, Gibson D, Gonzalez A, Gorlick K, Guo J, Hillmann B, Holmes S, Holste H, Huttenhower C, Huttley G, Janssen S, Jarmusch A, Jiang L, Kaehler B, Keefe C, Keim P, Kelley S, Knights D, Koester I, Kosciolek T, Kreps J, Lee J, Ley R, Liu Y-X, Loftfield E, Lozupone C, Maher M, Marotz C, Martin B, McDonald D, McIver L, Melnik A, Metcalf J, Morgan S, Morton J, Navas-Molina J, Orchanian S, Pearson T, Peoples S, Petras D, Pruesse E, Rivers A, Robeson M, Rosenthal P, Segata N, Shaffer M, Shiffer A, Sinha R, Spear J, Swafford A, Thompson L, Torres P, Trinh P, Tripathi A, Turnbaugh P, Ul-Hasan S, Vargas F, Vogtmann E, Walters W, Wan Y, Wang M, Warren J, Weber K, Willis A, Zaneveld J, Zhang Y, Zhu Q, Knight R, Caporaso G (2019). Reproducible, interactive, scalable and extensible microbiome data science using QIIME 2. Nature Biotechnology.

[ref-4] Bouvier T, Del Giorgio PA, Gasol JM (2007). A comparative study of the cytometric characteristics of High and Low nucleic-acid bacterioplankton cells from different aquatic ecosystems. Environmental Microbiology.

[ref-5] Browne HP, Forster SC, Anonye BO, Kumar N, Neville BA, Stares MD, Goulding D, Lawley TD (2016). Culturing of ‘unculturable’ human microbiota reveals novel taxa and extensive sporulation. Nature.

[ref-6] Brusa T, Canzi E, Pacini N, Zanchi R, Ferrari A (1989). Oxygen tolerance of anaerobic bacteria isolated from human feces. Current Microbiology.

[ref-7] Callahan BJ, Mcmurdie PJ, Rosen MJ, Han AW, Johnson AJA, Holmes SP (2016). DADA2: high-resolution sample inference from Illumina amplicon data. Nature Methods.

[ref-8] Caporaso JG, Lauber CL, Walters WA, Berg-Lyons D, Huntley J, Fierer N, Owens SM, Betley J, Fraser L, Bauer M, Gormley N, Gilbert JA, Smith G, Knight R (2012). Ultra-high-throughput microbial community analysis on the Illumina HiSeq and MiSeq platforms. ISME Journal.

[ref-9] Choo JM, Leong LE, Rogers GB (2015). Sample storage conditions significantly influence faecal microbiome profiles. Scientific Reports.

[ref-10] Chu ND, Smith MB, Perrotta AR, Kassam Z, Alm EJ (2017). Profiling living bacteria informs preparation of fecal microbiota transplantations. PLOS ONE.

[ref-11] David LA, Maurice CF, Carmody RN, Gootenberg DB, Button JE, Wolfe BE, Ling AV, Devlin AS, Varma Y, Fischbach MA, Biddinger SB, Dutton RJ, Turnbaugh PJ (2014). Diet rapidly and reproducibly alters the human gut microbiome. Nature.

[ref-12] Dethlefsen L, Huse SM, Sogin ML, Relman DA (2008). The pervasive effects of an antibiotic on the human gut microbiota, as revealed by deep 16S rRNA sequencing. PLOS Biology.

[ref-13] Emerson JB, Adams RI, Román CMB, Brooks B, Coil DA, Dahlhausen K, Ganz HH, Hartmann EM, Hsu T, Justice NB, Paulino-lima IG, Luongo JC, Lymperopoulou DS, Gomez-silvan C, Rothschild-Mancinelli B, Balk M, Huttenhower C, Nocker A, Vaishampayan P, Rothschild LJ (2017). Schrödinger’s microbes: tools for distinguishing the living from the dead in microbial ecosystems. Microbiome.

[ref-14] Faith JJ, Guruge JL, Charbonneau M, Subramanian S, Seedorf H, Goodman AL, Clemente JC, Knight R, Heath AC, Leibel RL, Rosenbaum M, Gordon JI (2013). The long-term stability of the human gut microbiota. Science.

[ref-15] Fofanov VY, Furstenau TN, Sanchez D, Hepp CM, Cocking J, Sobek C, Pagel N, Walker F, Chambers CL (2018). Guano exposed: Impact of aerobic conditions on bat fecal microbiota. Ecology and Evolution.

[ref-16] Fouhy F, Deane J, Rea MC, O’Sullivan Ó, Ross RP, O’Callaghan G, Plant BJ, Stanton C (2015). The effects of freezing on faecal microbiota as determined using miseq sequencing and culture-based investigations. PLOS ONE.

[ref-17] Friedman ES, Bittinger K, Esipova TV, Hou L, Chau L, Jiang J, Mesaros C, Lund PJ, Liang X, FitzGerald GA, Goulian M, Lee D, Garcia BA, Blair IA, Vinogradov SA, Wu GD (2018). Microbes vs. chemistry in the origin of the anaerobic gut lumen. Proceedings of the National Academy of Sciences.

[ref-18] Gilbert JA, Quinn RA, Debelius J, Xu ZZ, Morton J, Garg N, Jansson JK, Dorrestein PC, Knight R (2016). Microbiome-wide association studies link dynamic microbial consortia to disease. Nature.

[ref-19] Günther S, Koch C, Hübschmann T, Röske I, Müller RA, Bley T, Harms H, Müller S (2012). Correlation of community dynamics and process parameters as a tool for the prediction of the stability of wastewater treatment. Environmental Science & Technology.

[ref-20] Habtewold T, Duchateau L, Christophides GK (2016). Flow cytometry analysis of the microbiota associated with the midguts of vector mosquitoes. Parasites and Vectors.

[ref-21] Holý O, Chmelař D (2012). Oxygen tolerance in anaerobic pathogenic bacteria. Folia Microbiologica.

[ref-22] Huttenhower C, Gevers D, Knight R, Abubucker S, Badger JH, Chinwalla AT, Creasy HH, Earl AM, FitzGerald MG, Fulton RS, Giglio MG, Hallsworth-Pepin K, Lobos EA, Madupu R, Magrini V, Martin JC, Mitreva M, Muzny DM, Sodergren EJ, Versalovic J, Wollam AM, Worley KC, Wortman JR, Young SK, Zeng Q, Aagaard KM, Abolude OO, Allen-Vercoe E, Alm EJ, Alvarado L, Andersen GL, Anderson S, Appelbaum E, Arachchi HM, Armitage G, Arze CA, Ayvaz T, Baker CC, Begg L, Belachew T, Bhonagiri V, Bihan M, Blaser MJ, Bloom T, Bonazzi V, Paul Brooks J, Buck GA, Buhay CJ, Busam DA, Campbell JL, Canon SR, Cantarel BL, Chain PSG, Chen I-MA, Chen L, Chhibba S, Chu K, Ciulla DM, Clemente JC, Clifton SW, Conlan S, Crabtree J, Cutting MA, Davidovics NJ, Davis CC, DeSantis TZ, Deal C, Delehaunty KD, Dewhirst FE, Deych E, Ding Y, Dooling DJ, Dugan SP, Michael Dunne W, Scott Durkin A, Edgar RC, Erlich RL, Farmer CN, Farrell RM, Faust K, Feldgarden M, Felix VM, Fisher S, Fodor AA, Forney LJ, Foster L, Di Francesco V, Friedman J, Friedrich DC, Fronick CC, Fulton LL, Gao H, Garcia N, Giannoukos G, Giblin C, Giovanni MY, Goldberg JM, Goll J, Gonzalez A, Griggs A, Gujja S, Kinder Haake S, Haas BJ, Hamilton  HA, Harris EL, Hepburn TA, Herter B, Hoffmann DE, Holder ME, Howarth C, Huang KH, Huse SM, Izard J, Jansson JK, Jiang H, Jordan C, Joshi V, Katancik JA, Keitel WA, Kelley ST, Kells C, King NB, Knights D, Kong HH, Koren O, Koren S, Kota KC, Kovar CL, Kyrpides NC, La Rosa PS, Lee SL, Lemon KP, Lennon N, Lewis CM, Lewis L, Ley RE, Li K, Liolios K, Liu B, Liu Y, Lo C-C, Lozupone CA, Dwayne Lunsford R, Madden T, Mahurkar AA, Mannon PJ, Mardis ER, Markowitz VM, Mavromatis K, McCorrison JM, McDonald D, McEwen J, McGuire AL, McInnes P, Mehta T, Mihindukulasuriya KA, Miller JR, Minx PJ, Newsham I, Nusbaum C, O’Laughlin M, Orvis J, Pagani I, Palaniappan K, Patel SM, Pearson M, Peterson J, Podar M, Pohl C, Pollard KS, Pop M, Priest ME, Proctor LM, Qin X, Raes J, Ravel J, Reid JG, Rho M, Rhodes R, Riehle KP, Rivera MC, Rodriguez-Mueller B, Rogers Y-H, Ross MC, Russ C, Sanka RK, Sankar P, Fah Sathirapongsasuti J, Schloss JA, Schloss PD, Schmidt TM, Scholz M, Schriml L, Schubert AM, Segata N, Segre JA, Shannon WD, Sharp RR, Sharpton TJ, Shenoy N (2012). Structure, function and diversity of the healthy human microbiome. Nature.

[ref-23] Jellett JF, Li WKW, Dickie PM, Boraie A, Kepkay PE (1996). Metabolic activity of bacterioplankton communities assessed by flow cytometry and single carbon substrate utilization. Marine Ecology Progress Series.

[ref-24] Koch C, Günther S, Desta AF, Hübschmann T, Müller S (2013). Cytometric fingerprinting for analyzing microbial intracommunity structure variation and identifying subcommunity function. Nature Protocols.

[ref-25] Lebaron P, Servais P, Agogué H, Courties C, Joux F (2001). Does the high nucleic acid content of individual bacterial cells allow us to discriminate between active cells and inactive cells in aquatic systems?. Applied and Environmental Microbiology.

[ref-26] Longnecker K, Sherr BF, Sherr EB (2005). Activity and phylogenetic diversity of bacterial cells with high and low nucleic acid content and electron transport system activity in an upwelling ecosystem. Applied and Environmental Microbiology.

[ref-27] Maurice CF, Haiser HJ, Turnbaugh PJ (2013). Xenobiotics shape the physiology and gene expression of the active human gut microbiome. Cell.

[ref-28] Maurice CF, Turnbaugh PJ (2013). Quantifying and identifying the active and damaged subsets of indigenous microbial communities. Methods in Enzymology.

[ref-29] Maurice CF, Turnbaugh PJ (2013). Quantifying the metabolic activities of human-associated microbial communities across multiple ecological scales. FEMS Microbiology Reviews.

[ref-30] Morán XAG, Bode A, Suárez LÁ, Nogueira E (2007). Assessing the relevance of nucleic acid content as an indicator of marine bacterial activity. Aquatic Microbial Ecology.

[ref-31] Oliver DM, Haygarth PM, Clegg CD, Heathwaite AL (2006). Differential E. coli die-off patterns associated with agricultural matrices. Environmental Science & Technology.

[ref-32] Patel GB, Roth LA, Agnew BJ (2010). Death rates of obligate anaerobes exposed to oxygen and the effect of media prereduction on cell viability. Canadian Journal of Microbiology.

[ref-33] Peris-Bondia F, Latorre A, Artacho A, Moya A, D’Auria G (2011). The active human gut microbiota differs from the total microbiota. PLOS ONE.

[ref-34] Prest EI, Hammes F, Kötzsch S, Van Loosdrecht MCM, Vrouwenvelder JS (2013). Monitoring microbiological changes in drinking water systems using a fast and reproducible flow cytometric method. Water Research.

[ref-35] Proctor CR, Besmer MD, Langenegger T, Beck K, Walser JC, Ackermann M, Bürgmann H, Hammes F (2018). Phylogenetic clustering of small low nucleic acid-content bacteria across diverse freshwater ecosystems. ISME Journal.

[ref-36] Servais P, Casamayor EO, Courties C, Catala P, Parthuisot N, Lebaron P (2003). Activity and diversity of bacterial cells with high and low nucleic acid content. Aquatic Microbial Ecology.

[ref-37] Sträuber H, Müller S (2010). Viability states of bacteria-specific mechanisms of selected probes. Cytometry Part A.

[ref-38] The Integrative HMP (iHMP) Research Network Consortium (2019). The integrative human microbiome project. Nature.

[ref-39] Wang Y, Hammes F, Boon N, Chami M, Egli T (2009). Isolation and characterization of low nucleic acid (LNA)-content bacteria. ISME Journal.

[ref-40] Wang Y, Hammes F, De Roy K, Verstraete W, Boon N (2010). Past, present and future applications of flow cytometry in aquatic microbiology. Trends in Biotechnology.

[ref-41] Zimmermann J, Hübschmann T, Schattenberg F, Schumann J, Durek P, Riedel R, Friedrich M, Glauben R, Siegmund B, Radbruch A, Müller S, Chang HD (2016). High-resolution microbiota flow cytometry reveals dynamic colitis-associated changes in fecal bacterial composition. European Journal of Immunology.

[ref-42] Zimmermann J, Hübschmann T, Schattenberg F, Schumann J, Durek P, Riedel R, Friedrich M, Glauben R, Siegmund B, Radbruch A, Müller S, Chang HD (2016). High-resolution microbiota flow cytometry reveals dynamic colitis-associated changes in fecal bacterial composition. European Journal of Immunology.

[ref-43] Zubkov MV, Fuchs BM, Burkill PH, Amann R (2001). Comparison of cellular and biomass specific activities of dominant bacterioplankton groups in stratified waters of the celtic sea. Applied and Environmental Microbiology.

