# Supplementary material for: Effects of oxygen exposure on relative nucleic acid content and membrane integrity in the human gut microbiota
Source: PeerJ. 2021 Feb 3;9:e10602. doi: 10.7717/peerj.10602 (PMC7866891; doi:10.7717/peerj.10602)

Supplemental Figure 1: Median SSC values between HNA and LNA cells. Left: Acquired on the Calibur (N=28); Right: Acquired on the Canto (N= 33). Statistical significance was assessed as a paired t-test, error bars represent SD. *** P<0.0001

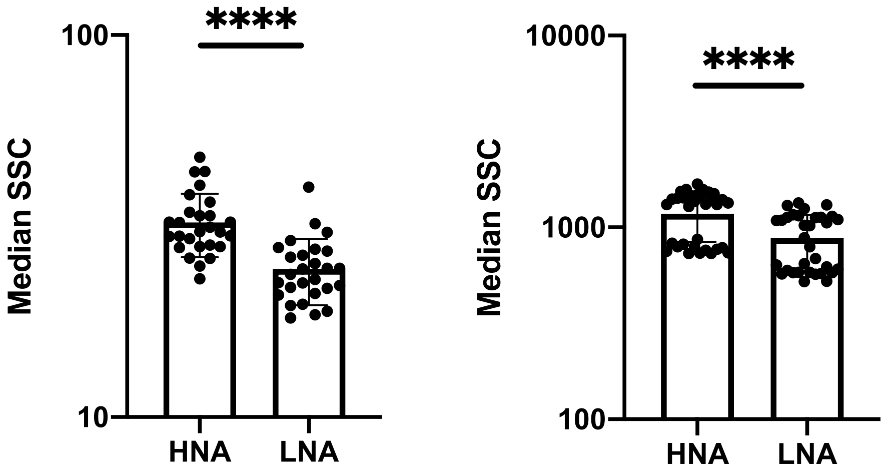

Supplement: Supplemental Information 7 — Left: Acquired on the Calibur (N=28); Right: Acquired on the Canto (N= 33). Statistical significance was assessed as a paired t-test, error bars represent SD. *** P<0.0001. [file peerj-09-10602-s007.pdf]
