# Supplementary material for: Effects of oxygen exposure on relative nucleic acid content and membrane integrity in the human gut microbiota
Source: PeerJ. 2021 Feb 3;9:e10602. doi: 10.7717/peerj.10602 (PMC7866891; doi:10.7717/peerj.10602)

Supplemental Figure 2: Absolute cell abundances in each oxygen exposure condition. Statistical significance was assessed with repeated measures one way ANOVA with Dunnet's multiple comparisons test. Paired samples are connected by a line between oxygen exposure conditions (n=8). * P<0.05

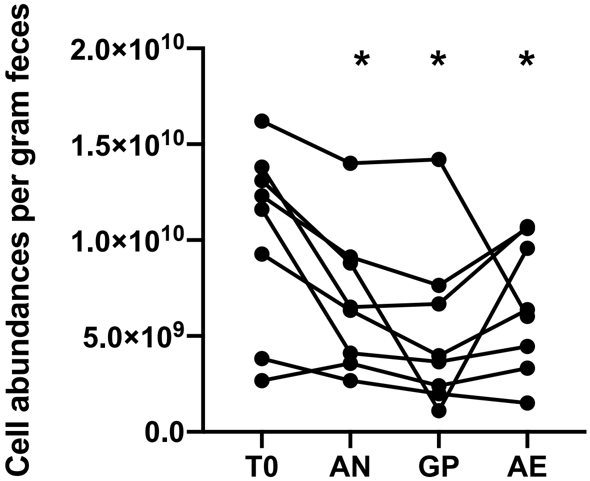

Supplement: Supplemental Information 8 — Statistical significance was assessed with repeated measures one way ANOVA with Dunnet’s multiple comparisons test. Paired samples are connected by a line between oxygen exposure conditions (n=8). * P<0.05. [file peerj-09-10602-s008.pdf]
