# Supplementary material for: Effects of oxygen exposure on relative nucleic acid content and membrane integrity in the human gut microbiota
Source: PeerJ. 2021 Feb 3;9:e10602. doi: 10.7717/peerj.10602 (PMC7866891; doi:10.7717/peerj.10602)

Supplemental Figure 3: The diversity of the initial community does not reflect the proportion of HNA or PI+ bacteria after oxygen exposure for 6 hours. Spearman correlation between the weighted UniFrac distances of the initial population to the proportion of HNA (left) or PI+ (right) bacteria after oxygen exposure (N = 10 individuals, 3 oxygen exposure conditions each, pairwise comparisons).

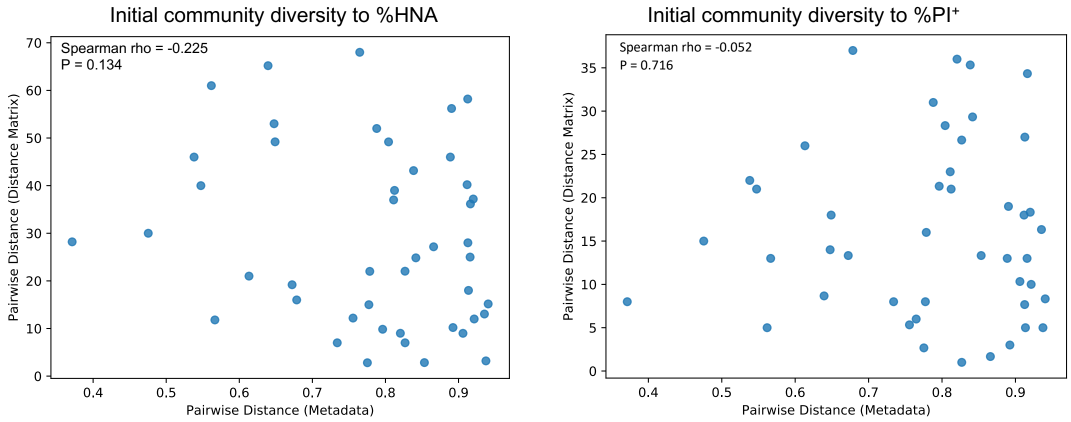

Supplement: Supplemental Information 9 — Spearman correlation between the weighted UniFrac distances of the initial population to the proportion of HNA (left) or PI+ (right) bacteria after oxygen exposure (N = 10 individuals, 3 oxygen exposure conditions each, pairwise comparisons). [file peerj-09-10602-s009.pdf]
